# Current evidence on the impact of the COVID-19 pandemic on paediatric endocrine conditions

**DOI:** 10.3389/fendo.2022.913334

**Published:** 2022-08-05

**Authors:** Margherita Gnocchi, Tiziana D’Alvano, Claudia Lattanzi, Giulia Messina, Maddalena Petraroli, Viviana D. Patianna, Susanna Esposito, Maria E. Street

**Affiliations:** Unit of Paediatrics, Department of Medicine and Surgery, University of Parma, and University Hospital of Parma, Parma, Italy

**Keywords:** lockdown, thyroid, vitamin D, obesity, precocious puberty, hyponatraemia, COVID-19, endocrinology

## Abstract

Severe acute respiratory coronavirus 2 (SARS-CoV-2) interacts with the host cells through its spike protein by binding to the membrane enzyme angiotensin-converting enzyme 2 (ACE2) and it can have a direct effect on endocrine function as ACE2 is expressed in many glands and organs with endocrine function. Furthermore, several endocrine conditions have features that might increase the risk of SARS-CoV-2 infection and the severity and course of the infection, as obesity for the underlying chronic increased inflammatory status and metabolic derangement, and for the possible changes in thyroid function. Vitamin D has immunomodulatory effects, and its deficiency has negative effects. Adrenal insufficiency and excess glucocorticoids affect immune conditions also besides metabolism. This review aims to analyze the rationale for the fear of direct effects of SARS-Cov-2 on endocrinological disorders, to study the influence of pre-existing endocrine disorders on the course of the infection, and the actual data in childhood. Currently, data concerning endocrine function during the pandemic are scarce in childhood and for many aspects definite conclusions cannot be drawn, however, data on properly managed patients with adrenal insufficiency at present are re-assuring. Too little attention has been paid to thyroid function and further studies may be helpful. The available data support a need for adequate vitamin D supplementation, caution in obese patients, monitoring of thyroid function in hospitalized patients, and confirm the need for an awareness campaign for the increased frequency of precocious puberty, rapidly progressive puberty and precocious menarche. The changes in lifestyle, the increased incidence of overweight and the change in the timing of puberty lead also to hypothesize that there might be an increase in ovarian dysfunction, as for example polycystic ovarian disease, and metabolic derangements in the next years, and in the future we might be facing fertility problems. This prompts to be cautious and maintain further surveillance.

## Introduction

Since December 2019 the world has been facing one of the most challenging health issues of the last centuries. Starting from Wuhan, in the Hubei province in China, a new unknown pathogen later identified as the severe acute respiratory coronavirus 2 (SARS-CoV-2) began to spread, causing an outbreak of severe atypical pneumonia (1).

In early January 2020 the causative virus of this new emerging illness was isolated and identified by the Chinese Center for Disease Control: the 2019 novel coronavirus was a positive-sense, single-stranded, enveloped RNA virus that belongs to β-coronavirus group, subfamily *Orthocoronavirinae* (family Coronaviridae) (2). Two other human β-coronaviruses have been responsible in recent years for severe lung infections: the severe acute respiratory syndrome (SARS-CoV) and the Middle East respiratory syndrome (MERS-CoV) coronavirus. Nevertheless, most coronaviruses known to date, such as Human coronaviruses 229E, NL63, OC43, and HKU1 are common agents of mild upper respiratory tract infections that mainly affect young children (3).

Despite COVID-19 infection tends to have a milder clinical course in the pediatric population with much less pulmonary disease and fewer deaths compared to adults, in some cases systemic involvement due to an abnormal hyperinflammatory response can be observed (4).

According to the WHO by October 2021 the incidence of COVID-19 infection in children under five years of age accounted for 2% of reported global cases, 7% between 5 to 14 years and 15% in adolescents and young adults (15 to 24 years) with a much lower mortality rate than in adults being the percentage of total deaths less than 0,5% of reported global deaths for subjects below the age of 25 years (5).

These data, however, have changed in recent months. In Italy, a significant increase of COVID-19 infection rate in the population 5-11 years with respect to other school-age children has been reported since October 2021, then followed by an overall increased incidence of the disease in older children and adolescents reported in the last weeks. Moreover, it is worth noting that the hospitalization rate in children under five years of age has strongly risen (> 10 hospitalizations per 1,000,000 inhabitants) with a less significant increase in the 16-19 age group in the last period (6). This must be kept in mind when considering the actual evidence in childhood as one must be aware that we still lack a complete figure of the effects of COVID-19 in the pediatric population, despite the recent increase of infection rates in this group. Moreover, given the higher mortality rate and overall worst outcome in adults suffering from chronic diseases, the question regarding a possible greater vulnerability in high-risk pediatric groups, such as children with underlying medical conditions, has arisen over time.

However, currently, strong evidence between specific chronic conditions and severe COVID-19 illness in children is limited, although it is well known that several medical conditions are known to be able to increase SARS-CoV-2 infection severity, such as inherited or acquired immunodeficiencies, immunosuppression, cancer, neurologic, endocrine and metabolic conditions. More generally, patients with multimorbidity affected by genetic diseases (i.e. Down syndrome), cardio-vascular diseases or other chronic conditions (i.e. asthma or chronic pulmonary disease) show an increased vulnerability to COVID-19 infection (7).

SARS-CoV-2 interacts with the host cells through its spike protein by binding to the membrane enzyme angiotensin-converting enzyme 2 (ACE2), an homolog of ACE, which represents the main viral receptor. The entry of SARS-CoV-2 in the host cell through ACE2 activates the STAT3/NF-kB pathway leading to the production of proinflammatory cytokines and chemokines that can cause a severe systemic hyperinflammation known as “cytokine storm”, responsible for acute respiratory distress syndrome (ARDS) and multi-organ failure (8).

Although SARS-CoV-2 mainly affects the upper and lower respiratory tract with symptoms ranging from flu-like symptoms to atypical pneumonia, ACE2 expression is not exclusive to the lungs and it may also involve the gastrointestinal tract, the kidneys, the cardio-vascular system and the brain, thus justifying the multi-organ involvement that is commonly observed in COVID-19 disease (9).

**Figure 1:**
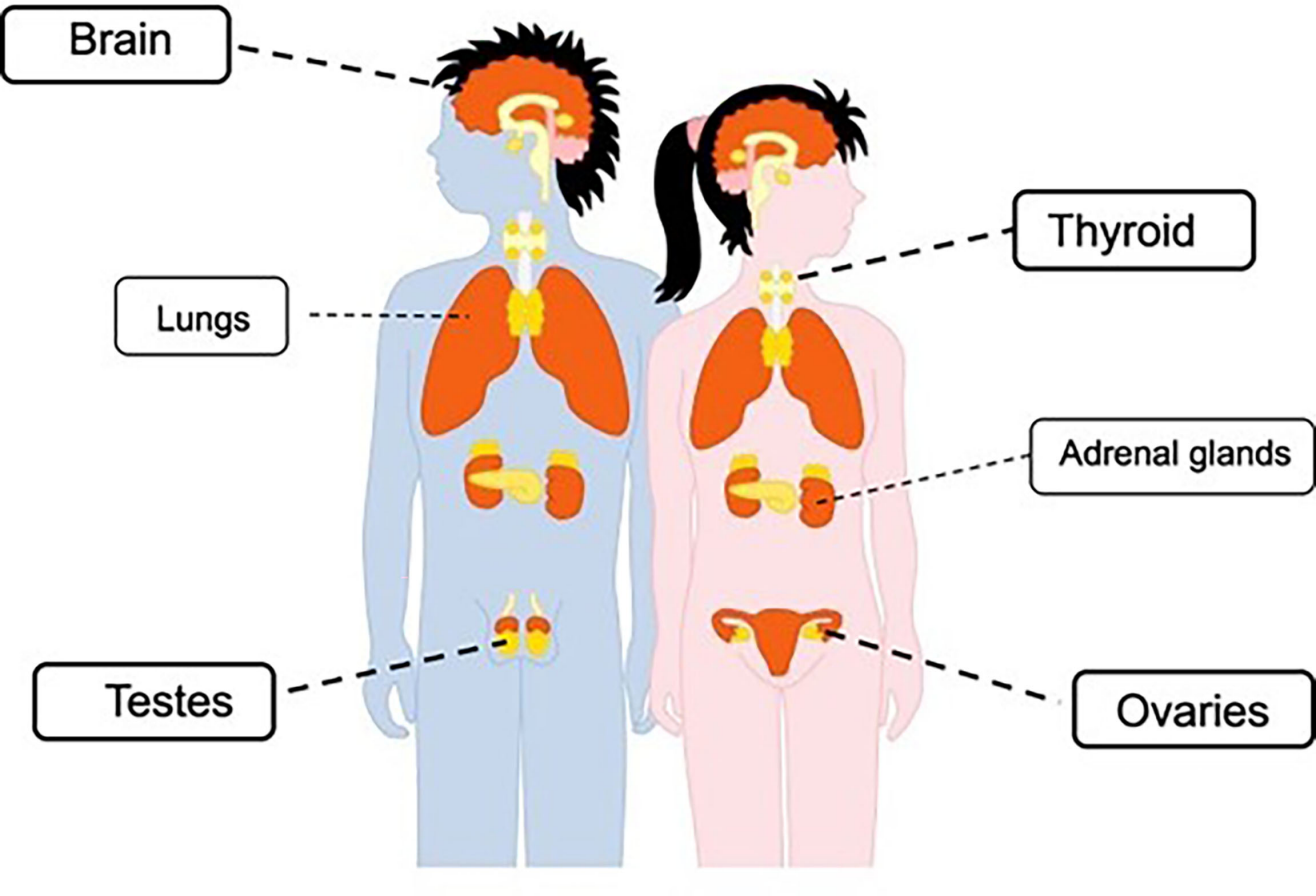
Representation of ACE2 expression in endocrine tissues.

Several endocrine tissues also exhibit ACE2, such as thyroid, the ovaries and the testis (the latter in particular with high levels of expression), for which the function of several glands may be affected by SARS-CoV-2 infection (**Figure 1**) (10, 11).

In addition, the treatment of endocrine diseases could be indirectly affected by difficulties arisen because of the pandemic. The scientific societies across the globe having endocrinology as a focus have provided recommendations since the beginning of the pandemic. The European Society for Pediatric Endocrinology (ESPE) highlighted that there was no evidence at that time that children affected by hypopituitarism (GHD, central hypothyroidism, diabetes insipidus, hypogonadotrophic hypogonadism or any combination of the aforementioned diseases) presented a higher risk of contracting the infection or experiencing a severe disease course (12). However, patients affected by hypopituitarism and secondary adrenal insufficiency potentially had a higher risk of undergoing an adrenal crisis during the SARS-COV2 infection as for any other infection (13).

Recommendations to continue current replacement therapy and to modify hydrocortisone replacement doses as indicated for all cases of intercurrent illness, doubling or tripling the dosage according to the severity of the clinical picture and conditions, also considering admission and parental hydrocortisone administration when necessary have been published by many scientific societies (14–16). Moreover, it was underlined to keep closely in touch with the reference centers (17).

This review aims to analyze the rationale for the fear of direct effects of SARS-Cov-2 on endocrinological disorders, to study the influence of pre-existing endocrine disorders on the course of the infection, and the actual data in childhood.

## Dysnatraemia, diabetes insipidus and pituitary function

​​SARS-COV 2 virus can access the central nervous system rapidly through the olfactory nerve (18), causing potential damage to hypothalamus and pituitary regions.

Previous studies that examined prevalence and outcomes of dysnatraemia in patients hospitalized with COVID-19 described a higher prevalence of hyponatremia than hypernatremia, but both these conditions were associated with an increased hospital length of stay, while the risk of in-hospital mortality was increased in patients with moderate or severe hypernatremia (19, 20).

Adequate treatment of hyponatremia requires immediate recognition of its etiology: blood tests are required to evaluate serum and urinary osmolality, serum concentrations of sodium, glucose, urea, creatinine, urate, cortisol, thyroxine and TSH (20).

One of the causes of hyponatremia highlighted by observational studies on adults with SARS-COV2 infection was posterior pituitary dysfunction, presenting mainly as the syndrome of inappropriate antidiuretic hormone secretion (SIADH) (21). In two adult patients described in the Literature, having severe COVID-19 and SIADH, the MRI scans showed T2/FLAIR hyperintensity of mammillary bodies, hypothalamus- pituitary swelling and upper globular hypophysis pedunculus (22). To the best of our knowledge, to date there still are no reports in children, however, considering the high incidence of gastroenterologic symptoms during SARS-COV2 infection in children (23) a concern for an increased risk of hypernatremia in patients affected by diabetes insipidus yet remains.

Diabetes insipidus (DI) is a rare disease characterized by polyuria (urine volume greater than 100–110 mL/kg/24 h until the age of 2 and 2000 mL/mq/24 h after the age of 2) caused by vasopressin secretion deficiency (central DI) or AVP resistance (nephrogenic DI) (24). Hyponatraemia (plasma sodium < 135 mmoL/L) is the major complication of chronic therapy with desmopressin; to prevent this condition electrolyte checks are recommended, but during the pandemic, considering the possibility of limited access to blood testing, expert recommendation was to avoid excessive water intake and delaying a dose of desmopressin until aquaresis occurs. While in patients known to have recurrent hyponatremia, one dose each week can be avoided, although greater polyuria may occur (25).

Patients with DI who develop respiratory complications of COVID-19 are at significantly increased risk of hypernatremic dehydration. However, experts agree to accept mild hypernatremia (<155 mmoL/L) rather than increase the risk of pulmonary oedema if it is corrected (25).. Hypernatremia and the related hyperosmolar state can cause physiologic alterations that are responsible for increased mortality. DI is associated also with a hypercoagulable state and low molecular weight heparin therapy is recommended in these cases, until eunatraemia is restored, as well as in patients with a hypercoagulable state during COVID 19 (26).

At present data in childhood in relationship with the pandemic are lacking but the same recommendations described in the guidelines published by the Society of Endocrinology in 2018 are valid for patients with DI with COVID-19 (27).

## Tumors of the hypothalamus and pituitary region

There are no data in the literature indicating that patients with pituitary and hypothalamic tumors

are more susceptible to infection with SARS-COV2. The only exception seems to concern corticotropic adenomas in patients with uncontrolled Cushing’s disease. These patients indeed show an increased risk of developing infections with subsequent increased risk of mortality (28, 29).

There are no reports of an increased severity of COVID-19 in patients with pituitary secreting adenomas or non-functioning adenomas that do not require steroid replacement therapy. However, it should be noted that risk factors may coexist in patients with pituitary tumors, which could adversely affect the course and the management of SARS-COV2 infections (30).

Another important factor to take into account is the possible diagnostic delay of tumors that may occur in the pandemic era because of the difficulty accessing primary care, necessary instrumentation and laboratory tests (31). This may stem both from the internal arrangements of hospitals which are committed to fighting the pandemic and providing attention and resources to critically infected patients, and from the patients own fear of exposing themselves to the risk of infection, with potentially serious consequences (32).

Fleseriu et al. summarized some diagnostic and management issues encountered during the pandemic, inherent to the diagnostic and therapeutic process of patients with pituitary tumors (33). These authors provided practical guidance to make the management of these patients as effective as possible in the era of COVID-19. In the case of patients in follow-up, routine care has generally been routinely provided worldwide through telemedicine avoiding face-to-face contact. Telemedicine has in fact greatly developed during the pandemic, it has enabled doctors to provide care to their patients, improving the management of chronic diseases (34). Outpatient visits could be postponed in most cases by 3-6 months, providing patients and caregivers with essential information to detect warning signs deserving immediate medical attention and clinical evaluation. It was suggested that laboratory investigations, if deemed relevant to clinical decisions, should be performed in laboratories close to home (or exceptionally performed at home in case of high-risk patients) and transferred to the specialist center (33). In Italy, to our knowledge this has happened in most parts of the country.

It was highlighted that patients with Cushing disease deriving from an adenoma or having comorbidities such as diabetes, hypertension and obesity should be reminded of their increased risk of severe infection (35–37).

In the case of newly diagnosed patients, it is necessary to collect a detailed history and perform an evaluation through a virtual or in-person visit, to identify any comorbidities and assess the timing within which to submit the patient to laboratory tests. In the case of pituitary tumors causing severe visual impairment, surgery should be the first therapeutic choice (38). As happened, testing the patients for SARS-Cov-2 infection before surgery is essential, especially in case of trans-sphenoidal surgery (considered a procedure with high risk of infection for which surgical antimicrobial prophylaxis is recommended) (39). If the test is positive, it would be recommended to postpone the surgery until the infectious picture is resolved. If this is not possible the operating team must carefully wear safety equipment (40).

These recommendations, including the use of telemedicine, have currently become routine practice.

## Thyroid gland

**Figure 2:**
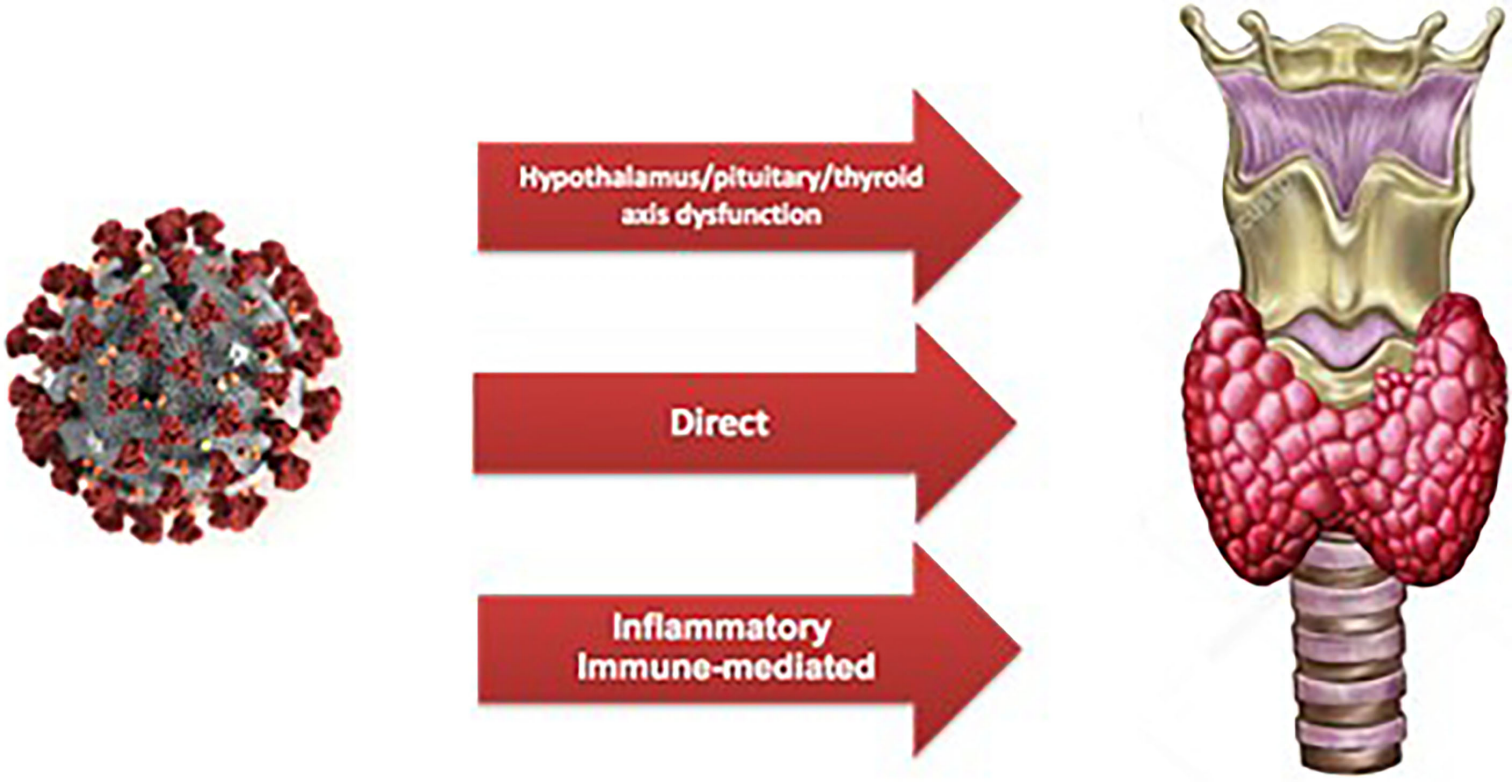
Representation of mechanisms of damage of SARS-CoV-2 to the thyroid gland.

The possible involvement of the thyroid gland in patients with COVID-19 infection has been widely investigated since the beginning of the pandemic, given the demonstrated ability of SARS-CoV-2 to attack thyroid follicular cells *via* ACE2 that is highly expressed in this tissue (10). Along with the direct mechanism of invasion of the thyroid tissue, indirect mechanisms represent another hypothesis of damage. In fact, acute hypothalamus/pituitary/thyroid axis dysfunction and abnormal activation of inflammatory factors and cytokines due to severe ongoing COVID-19 infection may be responsible for both acute dysthyroidism and the immune-mediated damage (**Figure 2**) (41).

A recent systematic review analyzed 27 documented and reported cases of subacute thyroiditis (17 case reports and 2 case series) likely related to SARS-CoV-2 infection. All reported patients were adults, the majority being women, with a median time of onset of the thyroid dysfunction 30 days after SARS-CoV-2 detection on swabs. Clinically, they showed typical symptoms of subacute thyroiditis (i.e. fever, fatigue, neck pain, palpitations) in association with thyrotoxicosis and elevated inflammatory markers in serum and ultrasonographic signs of thyroiditis. Interestingly, most patients were affected by mild to moderate COVID-19 disease with only three patients requiring hospitalization during the course of the infection. According to international guidelines for the treatment of subacute thyroiditis most patients were given corticosteroids with a progressive normalization of thyroid function and subsequent return to a euthyroid state. However, considering the large spread of the virus worldwide and the relatively lack of published data, at the moment it is only possible to consider that subacute thyroiditis represents a rare complication of SARS-CoV-2 infection with a clinical course that is similar to the one that is commonly observed when subacute thyroiditis is supposed to be triggered by other infections (42).

Conversely, other forms of thyroid dysfunction have been frequently reported in patients with severe COVID-19 disease, such as euthyroid sick syndrome or non-thyroidal illness syndrome (decreased fT3 but normal TSH levels) but also hypo- and hyperthyroidism as exacerbations of pre-existing disorders or *de-novo* diagnosis (43). Laboratory findings of low fT3 levels have been frequently observed in critical patients as a consequence of the abnormal hyperinflammatory state, representing an independent risk factor for COVID-19 severity. Therefore, considering the possible involvement of the thyroid during the course of COVID-19 infection and the heterogeneity of manifestations, routine assessment of thyroid function in those patients may be useful to detect thyroid abnormalities and start adequate treatment, thus improving the overall outcome (44, 45). It is important to point out, however, that all studies mentioned above concern adult patients and to date no reports regarding the association between COVID-19 and thyroid abnormalities in children are available.

On the other hand, well-managed thyroid disease does not appear to increase the risk of getting infected or develop severe COVID-19 illness both in children and adults, whereby international societies do not suggest a different management of children with pre-existing thyroid disease compared to healthy peers (46). Neither a pre-existing severe thyroid hormone deficiency (congenital hypothyroidism) if properly treated nor autoimmune diseases (Hashimoto’s thyroiditis or Graves’ disease) are associated with a weakened immune response or greater susceptibility to bacterial or viral infections. Anyhow, at the moment there is no evidence of an increased risk even in the case of poorly controlled thyroid dysfunction and no extra investigations are required, except for patients with thyrotoxicosis that may be at greater risk of complications because of the possible worsening of the thyroid storm in case of infection (47).

Parents of children who are being treated with antithyroid drugs such as methimazole should be advised about the possible bone marrow toxicity of these molecules: the onset of nonspecific symptoms such as fever, sore throat and cough may be the indicator of severe infection due to agranulocytosis that may present with a strong clinical overlap with COVID-19 infection, thus the necessity of prompt medical evaluation. Neither antithyroid drugs nor L-thyroxine are immunosuppressive agents and children who are administered with these medications do not present an increased risk of getting infected with SARS-CoV-2 (46).

However, the relative lower spread of SARS-CoV-2 infection among children may partially explain the current lack of significant correlation between COVID-19 disease and thyroid dysfunction in the pediatric population, with the need of further studies that may confirm the available recommendations.

## Adrenal insufficiency and hypercortisolism

Patients having primary adrenal insufficiency (i.e. CAH and Addison’s disease) are known to have a greater risk of life-threatening complications in case of infection because of the acute increased demand of glucocorticoids that may lead to adrenal crisis (48). Moreover, it has been demonstrated that adrenal failure makes these patients even more susceptible to common infections in general due to an impaired neutrophil and natural killer cell immune activity: this phenomenon could be explained by the altered circadian gene pattern expression of peripheral immune cells due the non-physiological delivery of glucocorticoids with the conventional replacement therapy (49). Different studies have indeed demonstrated that the use of daily modified-release preparations that mimic the physiological secretion of glucocorticoids helps restore an effective immune cell activity, thus improving hypoadrenal patients’ response to infections (50, 51). On the other hand, the replacement therapy itself, in consideration of the possible supraphysiologic doses of glucocorticoids chronically administered, may contribute to explain the increased risk of infections in patients affected by adrenal insufficiency.

Therefore, although at the moment there is no clear evidence of increased risks for COVID-19 infection in patients with primary adrenal insufficiency, all the reported data suggest the need for a careful management with close monitoring of these patients in case they get infected with SARS-CoV-2 because of their intrinsic condition of greater vulnerability to infections (52).

Therefore, as specified above, it has been repeatedly reminded that children presenting primary or secondary adrenal insufficiency should be treated following the general “sick day rule”: in case of mild to moderate infection (fever >38°C, bad cold or flu symptoms, diarrhea) their usual daily hydrocortisone dose should be immediately doubled or tripled if the fever is >39°C along with substantial oral hydration. In the case of a severe course of the infection or vomiting and subsequent inability of taking medications by mouth, urgent medical evaluation is mandatory in order to initiate parenteral administration of glucocorticoids and avoid a potentially life-threatening adrenal crisis. Conversely, asymptomatic patients should continue their daily replacement therapy without increasing the dosage (53). Moreover, the children who are being treated chronically with corticosteroids may develop tertiary adrenal insufficiency with a higher risk of getting infected by SARS-CoV-2 because of the subsequent iatrogenic immune impairment. Even though the level of suppression of the hypothalamus-pituitary-adrenal axis depends on the patient’s innate sensitivity and cannot be predicted, these patients must be considered to be at increased risk of a severe course of COVID-19 disease (16).

In line with these considerations, hypercortisolism due to an endogenous abnormal secretion (Cushing syndrome) may also increase the subject’s susceptibility to SARS-CoV-2 infection as a consequence of the immunosuppressive effect (54).

A few studies have analyzed both in Cushing syndrome and in different conditions characterized by adrenal insufficiency, the rate of infection and the course of COVID-19. In adults, during the first months in 2020 in Lombardy region in Italy, it has been shown that patients with active hypercortisolism were more prone to develop COVID-19 disease with a more severe clinical course with respect to the general population, thus identifying those affected by Cushing disease as a fragile population (55). In fact, it is worth considering that chronic cortisol excess leads to multiple comorbidities such as hypertension, hyperglycemia and obesity with an increase in visceral fat that have been widely associated with a greater risk of developing severe COVID-19.

Conversely, another observational case-control study also conducted during the early stages of the pandemic in Lombardy involving adult patients affected by adrenal insufficiency showed no significant differences in the incidence of SARS-CoV-2 infection, mortality rate, disease course and severity between patients having adrenal insufficiency and controls. However, all patients were on active follow-up and had been previously instructed on the possible risks and correct management of the infection (56).

At present few evidence is available because of the rarity of glucocorticoid-related diseases and pediatric scientific societies are currently collecting data, and at variance with the initial hypothesis with what had been initially hypothesized in relationship with adrenal insufficiency, possibly due to proper management, there does not seem to be an increased incidence of COVID-19 in these patients nor severe courses have been reported (14, 57).

One must also keep in mind that the initial phases of the pandemic have concerned few children and adolescents and currently the rate of vaccination is also increasing.

## Vitamin D deficiency, disorders of calcium homeostasis and parathyroid glands

Vitamin D has an undoubted role in the regulation of the immune system and deficient states have already been associated with an increased risk of developing upper respiratory tract infections and higher mortality rates in critically ill children (58, 59).

Indeed, most cells of the innate and adaptive immune system exhibit vitamin D receptors with the subsequent on-site production of 1,25(OH)_2_ vitamin D (the most active metabolite) that acts in an autocrine and paracrine way having immunomodulatory effects. Moreover, vitamin D receptors are also involved in the regulation of inflammatory cellular pathways, and their stimulation inhibits pro-inflammatory and pro-fibrotic response to tissue injury (60).

Based on this evidence, the potential role of vitamin D deficiency in COVID-19 patients’ outcome has been investigated since the beginning of the pandemic. Several randomized controlled trials in adults have actually confirmed the positive association between adequate vitamin D serum concentrations and a lower risk of getting infected by SARS-CoV-2 and developing severe complications (61). Moreover, important research studies that have collected and analyzed data coming from several countries have actually confirmed the positive correlation between vitamin D deficiency and COVID-19 infection and mortality rates in Asian countries, while in the European continent this correlation turned out to be significant only for COVID-19 related deaths with no impact on the total number of SARS-CoV-2 infections (62).

With regard to the pediatric population, only few studies are currently available concerning the effects of vitamin D deficiency on COVID-19 disease. A retrospective cohort study performed on hospitalized patients aged 0-18 years showed a significant relationship between low serum vitamin D concentrations, severe disease and elevated inflammatory markers: in particular, vitamin D levels were positively correlated with white cell blood count and negatively with C-reactive protein and fibrinogen levels (63, 64). However, the actual impact of vitamin D deficiency on COVID-19 infection is still under debate and further investigations are yet needed to clarify this matter. Meanwhile, considering vitamin D multiple skeletal and extraskeletal beneficial actions in association with its immunomodulatory effects, prophylactic vitamin D supplementation is highly recommended in the pediatric population as an easily modifiable risk factor that may protect from SARS-CoV-2 major complications (65).

Since several studies conducted in the pre-COVID era have demonstrated that vitamin D supplementation of deficient populations may help prevent infections, the prompt correction of hypovitaminosis with therapeutic dosages has become now mandatory and seems useful also in hospitalized patients with severe COVID-19 (66, 67).

Calcium is recognized to play an important role in viral infections, and hypocalcemia has been reported in many cases of adult patients with covid-19 and requiring admission to hospital (68, 69), and has been related also with a higher mortality risk (70). Although vitamin D deficiency is one of the main causes of hypocalcaemia, inappropriate levels of parathyroid hormone (PTH) are a contributing factor (71). Although there are no reports in childhood, recently two case reports evidence that SARS-CoV-2 infection can cause primary hypoparathyroidism and decompensate previous primary hypoparathyroidism independent of vitamin D levels (72, 73).

## Obesity

Obese people experience functional impairment and a major tendency to comorbidities, resulting in a greater susceptibility to infection from SARS-CoV-2 and a more severe course of COVID 19, with higher risk of developing a critical situation (74). A higher body mass index (BMI > 40 kg/m^2^) correlates with an increased risk for hospitalization, in particular in intensive care units, and for mortality, as previously highlighted for other respiratory viruses also by Fezeu et al. in their meta-analysis based on a systematic review of the Medline and Cochrane databases (75–77).

Gao et al. designed a prospective, community-based, cohort study and found that the risk of critical disease and death increased linearly with BMI for values above 23 kg/m^2^, mostly in people younger than 40 years and black. In detail, for a BMI above 23 kg/m^2^, every unit rise increases the risk for hospitalization (Hazard Ratio 1.09, 95% CI 1.08-1.10), intensive care need (Hazard Ratio 1.13, 95% CI 1.11-1.16) and death (Hazard Ratio 1.17, 95% CI 1.11-1.23) by COVID-19 disease (78).

The reason for the increased susceptibility is related to many factors. COVID-19 stimulates a cytokine storm, which worsens the subclinical basal inflammation associated with obesity. Inflammation then determines mechanisms leading to insulin resistance, hyperglycemic status and major thrombotic risk, establishing a vicious circle of harmful events (79).

Liu et al. have stated that the levels of circulating IL-6 and C-reactive protein may efficiently predict the severe course of the disease and the adverse outcome of patients affected by SARS-CoV-2 (80).

**Figure 3:**
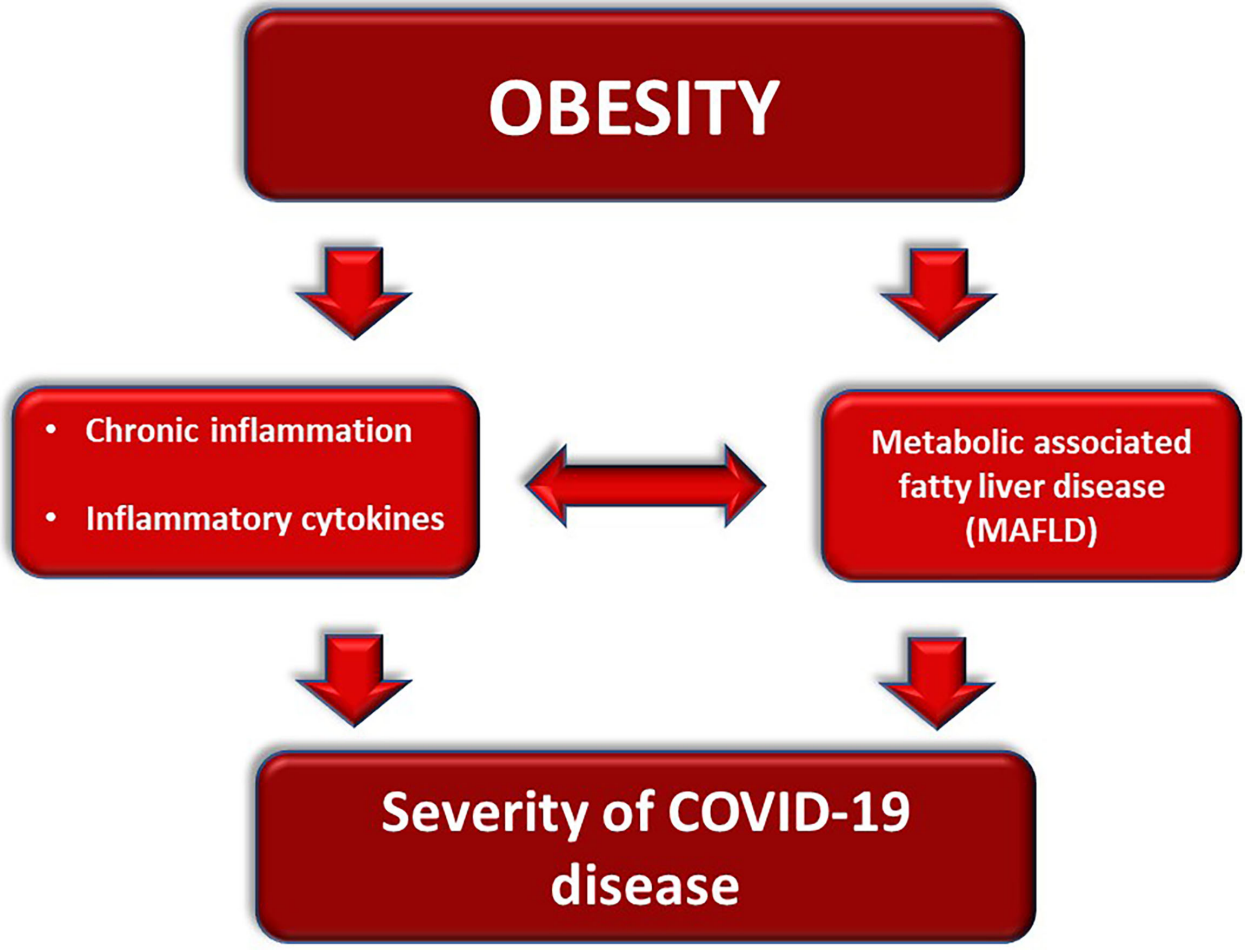
Obesity-related mechanisms worsening the course of COVID-19.

Actually, Gao et al. found that patients affected by SARS-CoV-2 infection and metabolic dysfunction associated with fatty liver disease (MAFLD) present an increased level of the inflammatory cytokine IL-6 compared to patients without MAFLD. Moreover, elevated IL-6 levels correlated with a greater risk of developing severe COVID-19, thus high IL-6 levels were associated with a major hazard for severe COVID-19 in patients with MAFLD than in those without (**Figure 3**) (81).

Within the frame of inflammation, the role of HMGB1 should also be recalled. Patients with increased serum concentrations of this NFkb activator and damage-associated molecular pattern protein and alarmin. It is well known to be associated with obesity, insulin resistance, and diabetes, and is involved with thrombosis-related diseases, sepsis, triggered beta amyloid accumulation in central nervous system, gene polymorphisms associated with hypertension, all associated with a severe course of COVID-19 or related risk factors (82).

The pathogenesis of the major severity of the disease could be explained by the virus-induced cytokine storm intensified by MAFLD that causes the secretion of inflammatory cytokines by the liver. Furthermore, COVID-19 could activate macrophages, resulting in the release of IL-6 with further worsening of the cytokine storm (83).

**Figure 4:**
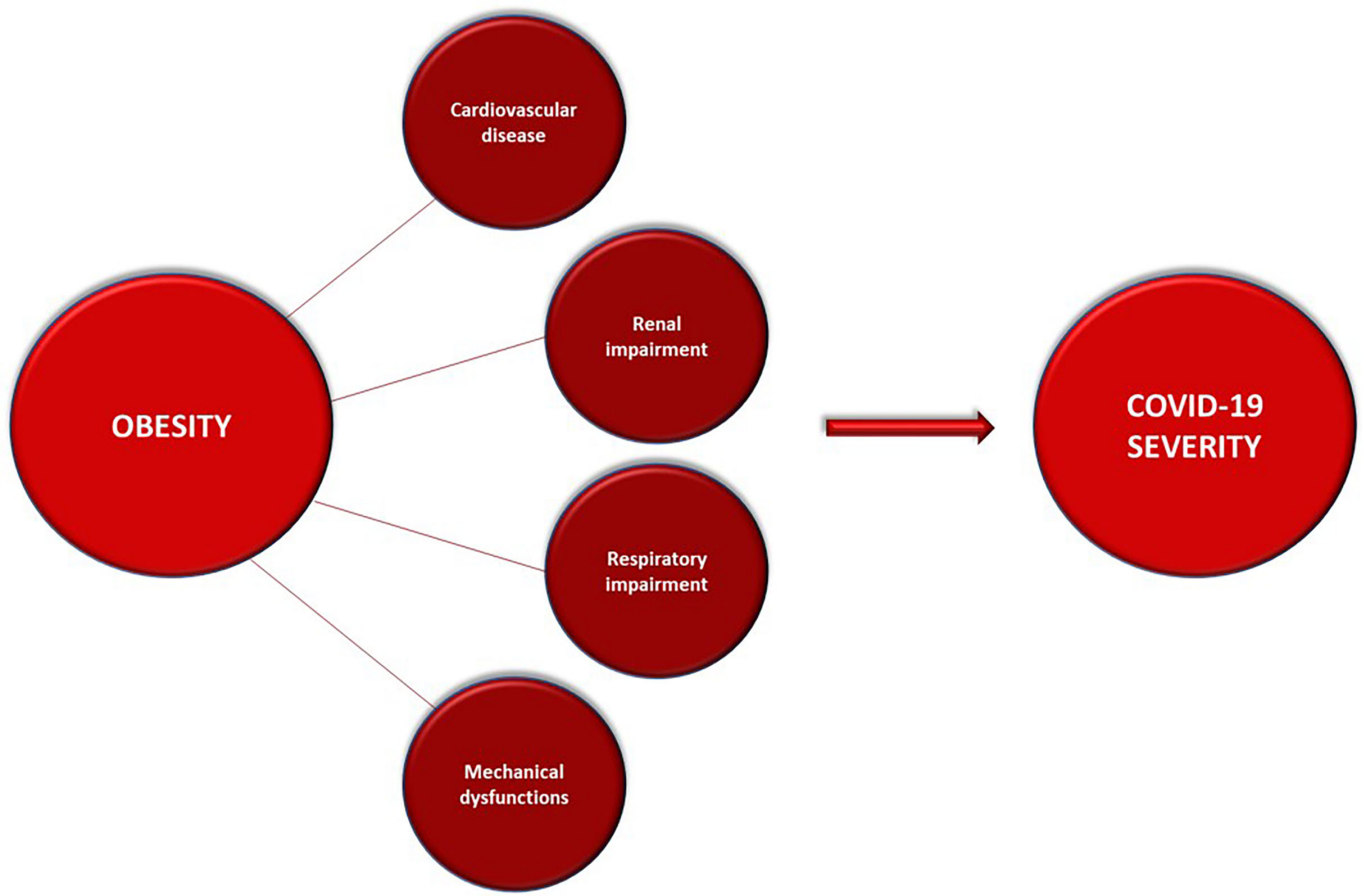
Obesity-related clinical complications that can underly and worsen the course of COVID-19.

Obesity leads to a wide range of clinical complications (**Figure 4**). Respiratory system compliance and the functional residual capacity are reduced, whilst abdominal pressure, oxygen expense and respiratory fatigue are augmented up to muscle exhaustion. Moreover, obstructive apnoea syndrome is most frequently experienced by obese people, making the airways management harder (84).

Obesity is a contributing cause of cardiovascular impairment, deriving from a higher blood volume leading to heart failure. It is inextricably linked to metabolic syndrome, as it indirectly influences dyslipidemia and blood pressure increasing the hazard for ischemic cardiopathy (85).

Kidney function is also impaired by obesity, which is directly linked to acute kidney injury; moreover, blood overload due to overweight leads to glomerular sclerosis and comorbidities as type 2 diabetes and hypertension are well-known risk factors for chronic kidney disease (86).

Nonetheless, hospitalized obese people experience mechanical and practical issues too: the management of these patients in intensive care unit is more difficult, as they develop ARDS more frequently, but intubation is challenging as well as proning; in addition, it is more difficult to cannulate peripheral vessels and central accesses are at higher risk of infection (87).

Thus, institutions should continue addressing prevention measures to obese people aiming to reduce the individual and population burden of the disease which has become increasingly more difficult during the pandemic (88).

Evidence confirms that we are facing a double pandemic, meaning both the spread of COVID-19 and obesity. These considerations are of considerable importance in light of the fact that the pandemic has deeply affected the population lifestyle. This is particularly evident for certain categories such as adolescents, among whom the prevalence of overweight has increased during the lockdown. On one hand, this may be due to physical inactivity, and on the other hand to the worsening of eating habits. These problems also result in psychological discomfort which is experienced by children and young people during this pandemic (89).

A high rate of changes in eating habits was recently shown in a study in 1,519 people to whom a specific questionnaire was administered. Specifically, a higher use of frozen food and an increased consumption of coffee and sweets, leading to weight gain was observed in the majority of participants. At the same time, a decrease in physical activity was also reported (90).

These feedbacks confirm an overall worsening of lifestyle that leads to psychological distress: children and adolescents experience a high risk of developing obesity during the pandemic, because they are facing new stressing situations. New routines have replaced the protective environments of school and family and social interactions, increasing stress and subsequently aggravating disparities, health problems, economical concerns and social issues (91).

The daily grind of both patients and healthcare professionals has suddenly changed, making it urge to find new tools and practices responding to current needs. Under this scenario, telemedicine has often answered to requirements of this new situation (92). Pecoraro et al. have evaluated the utility of telemedicine in the follow-up of obese patients by offering exercises, tutorials and games to play during the lockdown. At the end of this period, they measured anthropometric parameters (body mass index, waist circumference, fat mass and fat free mass) and found an improvement in body composition, with reduced fat mass (93). While considering the limitations of the short period and the small sample analyzed, this study paves the way for further research to improve patient status using technology.

## Central precocious puberty

The incidence of central precocious puberty (CPP) has been steadily increasing over the last century, with average age at menarche dropping from 17 years in the early-1800s to 13 years by the mid-1900s, with a further minor decline through the last three decades (94).

Such trend in age at pubertal onset – known as “secular trend of puberty” – recognizes both genetic and environmental factors which contribute to determine the timing and the *tempo* of puberty.

Following the COVID-19 outbreak in February 2020, a significant increase in the diagnoses of CPP, rapidly progressing puberty and precocious menarche has been reported by some paediatric Endocrinologists in Italy (95–97). Stagi *et al.* were the first to evaluate retrospectively the incidence of newly diagnosed CPP and the rate of pubertal progression in girls previously diagnosed with CPP between March and July 2020, and compared these data with medical records from the same period of time of the previous five years (2015-2019) (95). They found that the number of newly diagnosed CPP in 2020 was significantly higher than that in each year from 2015 to 2019 (37 vs 17.8 ± 1.3 patients, range from 16 to 19 cases/same period/year), along with a significantly younger chronological age at stage B2 onset/CPP diagnosis and a more advanced Tanner stage at CPP diagnosis. Moreover, they described a greater number of patients with previously diagnosed CPP moving from a slowly progressing puberty to a rapidly progressing puberty following the Italian lockdown, in comparison with the previous five years (12 vs 2.2 ± 0.4 patients, range from two to three cases/same period/year). Similar findings were reported by Verzani et al. who retrospectively analyzed medical records of their outpatient clinic from March to September 2020, comparing them with the same interval of time in 2019, and reported a significant increment of CPP in girls, observing no differences for boys (96).

A more recent Italian retrospective study has likewise reported a significantly increased incidence of both suspected and confirmed CPP in girls from March to September 2020, in comparison with the same period in 2019 (98). No difference was found in the anthropometric and hormonal parameters between 2019 and 2020, whilst girls diagnosed with CPP in 2020 showed an increased use of electronic devices paired with an overall more sedentary lifestyle.

The literature currently lacks studies enlisting larger samples of CPP patients. Moreover, data from the experience of Pediatric Endocrinology centers abroad are very scarce. A single-center retrospective study carried out in Turkey confirmed the Italian findings, reporting an increased incidence of idiopathic CPP in girls from April 2020 to March 2021, following their national lockdown, when compared with the same period of time in the previous three years (2017-2019) (99). Conversely, they did not observe significant differences in the frequency of rapidly progressing puberty and obesity before and after the COVID-19 pandemic.

When it comes to the possible underlying causes of this phenomenon, both direct and indirect mechanisms – secondary to the lockdown and the deep changes in our society and everyday life – must be taken into account. Possible factors contributing to CPP and rapidly progressing puberty are reported in **Table 1**.

**Table 1:** Possible factors contributing/causing precocious puberty and rapidly progressive puberty.

Direct mechanisms	Indirect mechanisms
•Effects of SARS-CoV-2 on the CNS through: - ACE-2 receptor - cytokine storm syndrome - hematogenous route - olfactory route	Increased food consumption Decreased physical activity Increased BMI Increased use of electronic devices Changes in sleep habits and changes in melatonin secretion Increased mental stress and emotivity Possible increased exposure to some endocrine disruptors due to increased indoor life

CNS, central nervous system.

As for the direct effects of the SARS-CoV-2 infection, Nagu et al. very well described the different routes for invasion of the CNS, which include the binding to the ACE-2 receptors – diffusely expressed on the endothelial cells of capillaries –, a cytokine storm syndrome, a hematogenous route and a olfactory route (100). Intriguingly, the olfactory tract shares its embryonic origin with the Gonadotropin Releasing Hormone (GnRH) neurons in the hypothalamus, which pulsatile activation determines the onset of puberty (97). Moreover, a recent work found a positive correlation between the volume of the olfactory bulb and the incidence of CPP, suggesting that the olfactory tract plays a major role in determining the timing of puberty (101). In addition, the olfactory route is very abundant of GABAergic neurons, which contribute to determine the timing of puberty (102).

As for the indirect effect of the COVID-19 pandemic, the deep changes in our society and everyday life must be carefully addressed given the profound impact that they could have on the timing and *tempo* of puberty. The adjustment in food habits, the reduced access to physical activities, the overall increase in BMI, the increased use of digital devices, the worsened quality of sleep and the global emotional stress that children of all ages have undergone in the last two years could all be contributing factors to the increased risk of CPP.

At present, we lack studies which aim to analyze the role of each one of these mechanisms in the increasing trend which has been observed. Further investigations should be pursued in order to better investigate this phenomenon and understand the triggering role of each single environmental factor on the timing and the *tempo* of pubertal onset.

## Conclusions

Current findings related to COVID-19 and pediatric endocrine conditions are reassuring in terms of rate of infection and severity of the disease in almost all properly managed conditions. The data support a need for adequate vitamin D supplementation, caution in obese patients, monitoring of thyroid function in hospitalized patients, and confirm the need for an awareness campaign for the increased frequency of precocious puberty, rapidly progressive puberty and precocious menarche. The changes in lifestyle, the increased incidence of overweight and the change in the timing of puberty lead also to hypothesize that there might be an increase in the following years of ovarian dysfunction as polycystic ovarian disease, and metabolic derangements, therefore requiring a continuous surveillance.

## Author contributions

MES conceived, designed and supervised this study. MG, TD, CL and GM performed the literature review and wrote the first draft of the manuscript. MES, MP and VP contributed to the interpretation of the results and in writing the manuscript. MES and SE provided scientific contributions and critically revised the paper. All the authors read, revised, and approved the final manuscript.

## Conflict of interest

The authors declare that the research was conducted in the absence of any commercial or financial relationships that could be construed as a potential conflict of interest.

## Publisher’s note

All claims expressed in this article are solely those of the authors and do not necessarily represent those of their affiliated organizations, or those of the publisher, the editors and the reviewers. Any product that may be evaluated in this article, or claim that may be made by its manufacturer, is not guaranteed or endorsed by the publisher.
